# Basic epidemiological parameter values from data of real-world in mega-cities: the characteristics of COVID-19 in Beijing, China

**DOI:** 10.1186/s12879-020-05251-9

**Published:** 2020-07-20

**Authors:** Xiaoli Wang, Yang Pan, Daitao Zhang, Lijuan Chen, Lei Jia, Xinyu Li, Peng Yang, Quanyi Wang, C. Raina Macintyre

**Affiliations:** 1 Beijing Center for Disease Prevention and Control, Beijing, China; 2 Beijing Research Center for Preventive Medicine, Beijing, China; 3 School of Public Health, Capital Medical University, Beijing, China; 4 Kirby institute, Faculty of Medicine, the University of New South Wales, Sydney, NSW 2052 Australia; 5 College of Public Service & Community Solutions and College of Health Solutions, Arizona State University, Tempe, 85281 USA

**Keywords:** COVID-19, Epidemiology, Transmissibility, Secondary attack rate

## Abstract

**Background:**

With the spread of SARS-CoV-2 worldwide, understanding the basic epidemiological parameter values of COVID-19 from real-world data in mega-cities is essential for disease prevention and control.

**Methods:**

To investigate the epidemiological parameters in SARS-CoV-2 infected cases in Beijing, we studied all confirmed cases and close contacts in Beijing from Jan 1st to Apr 3rd 2020. The epidemiological and virological characteristics of SARS-CoV-2 were analyzed.

**Results:**

A total of 602 cases were positive for SARS-CoV-2, including 585 confirmed patients and 17 asymptomatic infections. The imported cases were mainly from Wuhan initially and then from abroad. Among 585 confirmed case-patients, the median age was 39 years old. The mean incubation period was 6.3 days. The secondary attack rate among households was higher than social contacts (15.6 vs 4.6%). The secondary attack rate of healthcare workers (HCWs) was higher than non-HCWs’ (7.3 vs 4.2%). The basic reproduction number was 2.0, and the average serial interval was 7.6 days. No significant genetic variant was identified.

**Conclusions:**

The transmissibility of SARS-CoV-2 was relatively high, especially among households and from HCWs, which draws specific public health attention. So far, no evidence of widespread circulation of SARS-CoV-2 in communities in Beijing was found.

## Background

In December 2019, a new beta coronavirus (CoV) was first detected in Wuhan, Hubei Providence of China, causing clusters of unknown pneumonia patients. It spread rapidly throughout Hubei and then to other provinces and abroad. The World Health Organization (WHO) declared the outbreak a public health emergency of international concern on January 30th, 2020 [1]. The novel coronavirus has been named SARS-CoV-2 by the International Committee on Taxonomy of Viruses and the disease it causes has been named “coronavirus disease 2019” (COVID-19) [2]. As of Apr 12th, 2020, the global number of confirmed cases of COVID-19 has surpassed 1,600,000, including more than 100,000 deaths due to acute respiratory failure or other related complications [3].

Currently, the minority of the population of China has been infected, which leaves the majority of China susceptible to infection. Beijing, the capital of China, which has a population of 21 million residents including 8 millions of migrants at particular risk. Since the first case detected in Beijing on Jan 19th, the number of cases imported from Wuhan increased soon. To prevent further spread, the Chinese government announced the lockdown (forced quarantine or in-and-outflow restriction) of Wuhan City on Jan 23rd before the upcoming spring festival during which massive population movements were expected to take place. Since it is of vital importance to get knowledge of basic epidemiological parameter values from data of real-world in mega-cities, we report the epidemiological and virological findings of 602 cases with SARS-CoV-2 infection and the outbreak response conducted in Beijing from Jan 1st to Apr 3rd 2020.

## Methods

### Outbreak response

Protocols for COVID-19 diagnosis and treatment, surveillance, epidemiological investigation, management of close contacts, and laboratory testing were formulated, and relevant surveillance activities and epidemiological investigations conducted. All individuals with suspected SARS-CoV-2 infection were isolated in designated hospitals for treatment. The epidemiological investigation, contact tracing, and quarantine were conducted by the Centers for Disease Prevention and Control (CDCs) at district level or municipal level. Close contacts were defined based on the protocol released by National Health Commission of the People’s Republic of China (NHCPRC). Without any efficient personal protective equipment, a person who had contact within one meter with a confirmed case after onset of symptoms was defined as close contact. The definition changed on Feb 10th in accordance with the WHO guidelines [4], from the onset to 4 days prior to symptoms, given evidence of presymptomatic transmission. Close contacts were required to be quarantined at home or in designated sites for cases who have no separate room or imported cases from overseas for medical observation till 14 days after the last exposure. Besides traditional contact tracing, big data and artificial intelligence (AI) were used to strengthen contact tracing. For enhanced surveillance to detect COVID-19 in influenza-like illness (ILI) cases, all samples from ILI cases reported in routine influenza virological surveillance system [5] were tested for SARS-CoV-2 using real-time reverse transcription-polymerase chain reaction (Real-time RT-PCR) since January 28th.

### Case definitions

The case definitions of suspected and confirmed cases of COVID-19 were based on the protocol released by the NHCPRC [6]. Some modifications in case definitions were made according to recent studies in China. The definition modifications of suspected cases and confirmed cases are shown in Supplementary Table 1. Among all confirmed cases, mild cases were defined cases without evidence of pneumonia, and moderate cases as those with pneumonia but no oxygen therapy required. Severe cases were defined as those with dyspnea, respiratory frequency ≥ 30/min, blood oxygen saturation ≤ 93%, PaO_2_/FiO_2_ ratio < 300, and/or lung infiltrates > 50% within 24–48 h. Critical cases were those that exhibited respiratory failure, septic shock, and/or multiple organ dysfunction/failure.

Asymptomatic laboratory-confirmed cases were defined a person who were positive for SARS-CoV-2 while showing no clinical symptoms and signs.

### Data collection

The demographic and epidemiological information, clinical symptoms and outcomes were obtained using a standardized questionnaire by interviewing patients and/or their family members/relatives, attending doctors and other health care providers, supplemented by patient medical records [7]. All data were checked by two public health professionals.

### Laboratory testing

Upper and lower respiratory tract specimens, urine, blood, stool, and sputum specimens were obtained from patients. For all RNA extractions, RNA was extracted from 200 μL of sample and eluted in 90 μL elution buffer by KingFisher Flex Purification System (Thermo Fisher, USA). The real-time RT-PCR assay was performed by TaqMan Fast Virus 1-Step Master Mix (ThermoFisher) in ABI 7500 fast system (ABI, USA). 0.5 μM of forward primer, 0.5 μM of reverse primer, 0.25 μM of probe, and 5 μl of RNA sample were mixed in a 25 μL monoplex quantitative RT-PCR reaction. The primer and probe were generated following the national guideline [7], and the reaction condition was set according to the manufacturer’s protocol. Cases testing positive for both target genes (open reading frame 1ab and nucleocapsid protein) were determined as laboratory-confirmed cases. Meanwhile, commercial kit (Bio-germ Inc., Shanghai, China and ABT Inc., Beijing, China), which were approved by China Food and Drug Administration (CFDA), were also used in testing. For those cases tested by a commercial kit, the results were determined following the manufacturer’s instructions. Upper and lower respiratory tract specimens from confirmed cases were used for viral genome sequencing. Next-generation sequencing (NGS) was achieved based on a metagenomics strategy followed by Illumina sequencing (Illumina Inc., San Diego, CA, USA). Output data were assembled by a viral genome-targeted assembly pipeline with a homology search E-value of 1e^− 10^. Full genome sequences used in the current study are available on request. Sequence alignment was achieved by MAFFT [8]. The Neighbor-Joining (N-J) method with a bootstrap of 1000 was used for phylogenetic analysis.

### Statistical analysis

We used descriptive statistical methods to analyze the epidemiological characteristics of confirmed cases with SARS-CoV-2 infection. Monte Carlo approach was applied to simulate 1000 times and estimate the average incubation period and its confidential interval. Data analysis was performed using SPSS statistical software package version 20.0 (IBM SPSS Inc., Chicago, IL, USA) and GraphPad Prism version 7.0 (GraphPad Software., San Diego, CA, USA). All statistical tests were 2-sided, and statistical significance was set at *P* value less than 0.05.

### Ethics approval

Data collection and analysis of cases and close contacts were determined by the NHCPRC to be part of a continual public health outbreak investigation, and as such was granted exemption from the institutional review board.

## Results

### Epidemiological characteristics of confirmed cases

From Jan 1st 2020 to Apr 3rd 2020, respiratory specimens from 7432 suspected cases were tested for SARS-CoV-2, of which 602 (8.1%) were positive. Among the laboratory-confirmed SARS-CoV-2 infections, 585 were confirmed case-patients with symptoms and signs and 17 were asymptomatic COVID-19 cases. The proportion of asymptomatic infection was 2.8% (95% CI: 1.5–4.2).

**Fig. 1:**
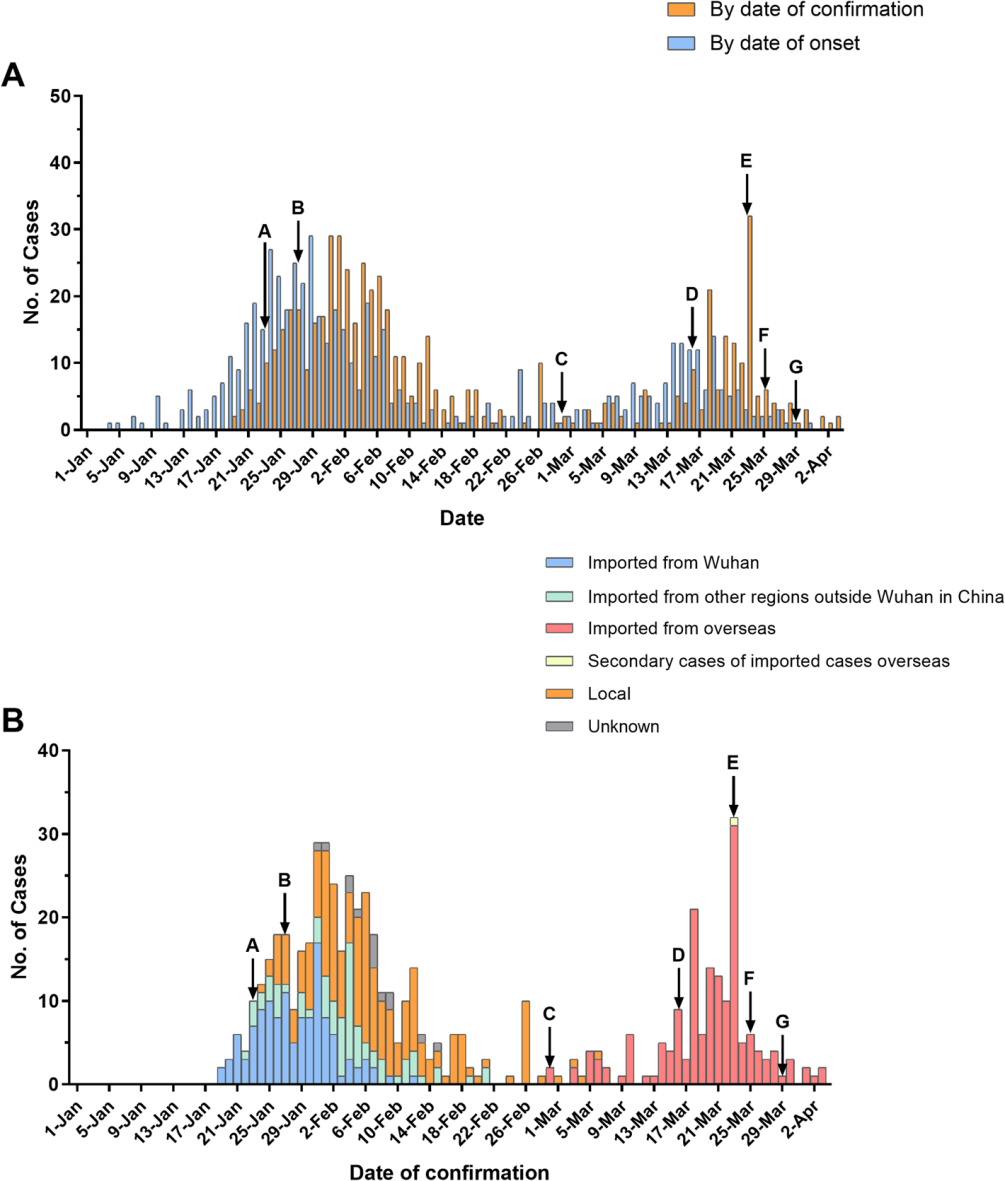
Epidemiologic Characteristics of 585 confirmed cases with SARS-CoV-2 infection. Note: Local cases include the secondary cases of imported case outside in China, excluding secondary cases of imported case from abroad. **a**: Wuhan was locked down on Jan 23rd. **b**: First fatal cases occurred on Jan 27th. **c**: First imported case from overseas identified on Feb 29th. **d**: Xiaotangshan Hospital launched for imported cases from overseas on Mar 16th. **e**: Nine cities designated as points of first entry for international passenger flights bound for Beijing on Mar 23rd. **f**: All international passengers arriving in Beijing must be tested and quarantined at designated facilities on Mar 25th. **g**: The number of imported cases from overseas in Beijing ranked first in China on Mar 29th

The epidemic curve in Beijing by date of confirmation and onset is shown in Fig. 1a. The first case was confirmed on Jan 19th. The number of infection rapidly grew to Jan 30th and peaked between Jan 31st and Feb 1st. The infections steadily declined between Jan 30th and Feb 18th. However, the number of reported cases fluctuated due to a local cluster (14 cases) that occurred in a company on Feb 25th in Beijing and imported cases from abroad.

Since the first imported case from Iran on Feb 29th, the number of cases from overseas increases rapidly. To effectively treat and manage these cases, Xiaotangshan Hospital was launched on Mar 16th. On Mar 23rd, nine cities were designated as the first entry sites for international passenger flights bound for Beijing. To detect more potential cases, all international passengers arriving in Beijing were required to be tested and quarantined at designated facilities on Mar 25th. The number of imported case from overseas in Beijing ranked first in China on Mar 29th. As of Apr 3rd, a total of 169 cases had been identified in Beijing. However, only 4 local cases were identified at the same time. And besides one local case was related to one imported case from abroad, no local cases had been reported for 27 days.

As shown in Fig. 1b, among 585 confirmed case-patients, 124 (21.2%) were imported from Wuhan, 76 (13.0%) from other regions of China, 169 (28.9%) from overseas, 1 (0.2%) was a secondary case of imported cases from overseas, 201 (34.4%) were local cases and 14 (2.4%) were under investigation. Among the 169 cases from overseas, most were from the United Kingdom (55), Spain (47), Italy (18), the United States of America (17) and France (7). The epidemic in Beijing has been undertaken in two main phases. The first phase started on Jan 19th when the first two cases were confirmed and ended on Feb 28th. The second phase started on Feb 29th when the first imported case from overseas was identified. At the beginning of the first phase (before Feb 1st), the majority of confirmed cases were imported from Wuhan (61.0%, 97/159), while the proportion of imported cases from Wuhan had decreased since Feb 1st (1 week after the lockdown of Wuhan) (16.1%, 27/168) (*χ*^*2*^ = 70.065, *P* < 0.001). At the beginning of the second phase (before Mar 6th when the last local cases identified), imported cases from overseas and local cases coexisted, and then the imported cases from overseas were dominated.

**Fig. 2:**
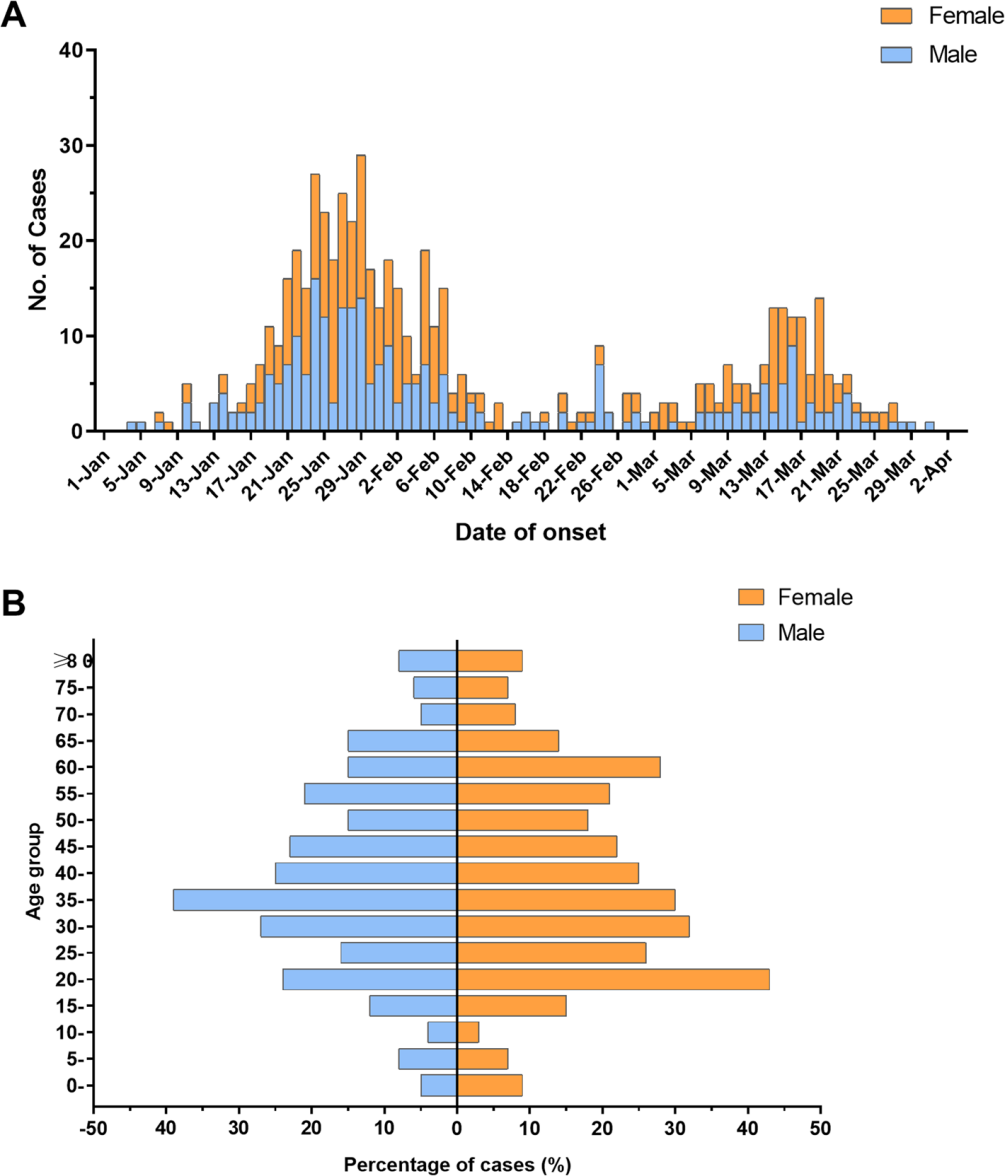
The age and sex distribution of 585 confirmed case-patients with SARS-CoV-2 infection

Of 585 confirmed case-patients, 268(45.8%) cases were male. The male-to-female ratio was 0.9:1. The incidence of males was slightly lower than females, 2.5/100,000 and 2.9/100,000, respectively (Fig. 2). The median age of confirmed cases was 39 years old (range, 0.5 to 94; interquartile range, 27 to 56). About 19% (114/585) were 60 or above, and 7.9% (46/585) were children < 18 years old, among which 34.8% (16/46) were children under 5 years old (Table 1). A total of 17 (2.9%) cases aged 80 years or above. The incidence rate of the population 60 years old or above (6.9/100,000) was the highest compared to the other three groups, followed by the 18–59 years old group (5.6/100,000), the 5–17 years old group (0.5/100,000), and children under 5 years old (0.3/100,000).

**Table 1:** Epidemiologic Characteristics of 585 confirmed case-patients with SARS-CoV-2 Infection in Beijing, China

Characteristic	Csese (*n* = 585) No./total no.(%)
**Sex**	
Male	268/585 (45.81)
Female	317/585 (54.19)
**Age group**	
0-	16/585 (2.74)
5-	30/585 (5.13)
18-	427/585 (72.99)
60-	112/585 (19.15)
**Imported cases**	368/585 (62.91)
**Exposure history**	
Huabei Seafood Wholesale Market	0/585 (0.00)
History of residence or travel	368/585 (62.91)
History of residence or travel in Wuhan	124/585 (21.20)
History of residence or travel in other regions outside Wuhan in China	76/585 (12.99)
History of residence or travel	169/585 (28.89)
Contact with confirmed or suspected cases	201/585 (34.36)
Contact with confirmed or suspected cases from abroad	1/585 (0.17)
Under investigation	14/585 (2.39)
**Health care worker**	17/585 (2.91)
**Case of severity**	
Mild	212/585 (36.24)
Moderate	291/585 (49.74)
Severe	66/585 (11.28)
Critical	16/585 (2.74)

The median age of 46 children< 18 years was 7 years old (interquartile range, 3 to 13). The sex ratio was 0.8:1. The proportion of asymptomatic infection among children< 18 years and adults was 4.2 and 2.7% (*χ*^*2*^ = 0.343, *P* = 0.558). Among 585 confirmed case-patients, 17 cases (2.9%) were healthcare workers (HCW). Epidemiological investigations suggested that 7 cases were infected due to health care activities and the remaining 10 were infected due to close contact with household cases rather than in a health care setting according to data from epidemiological investigation.

**Fig. 3:**
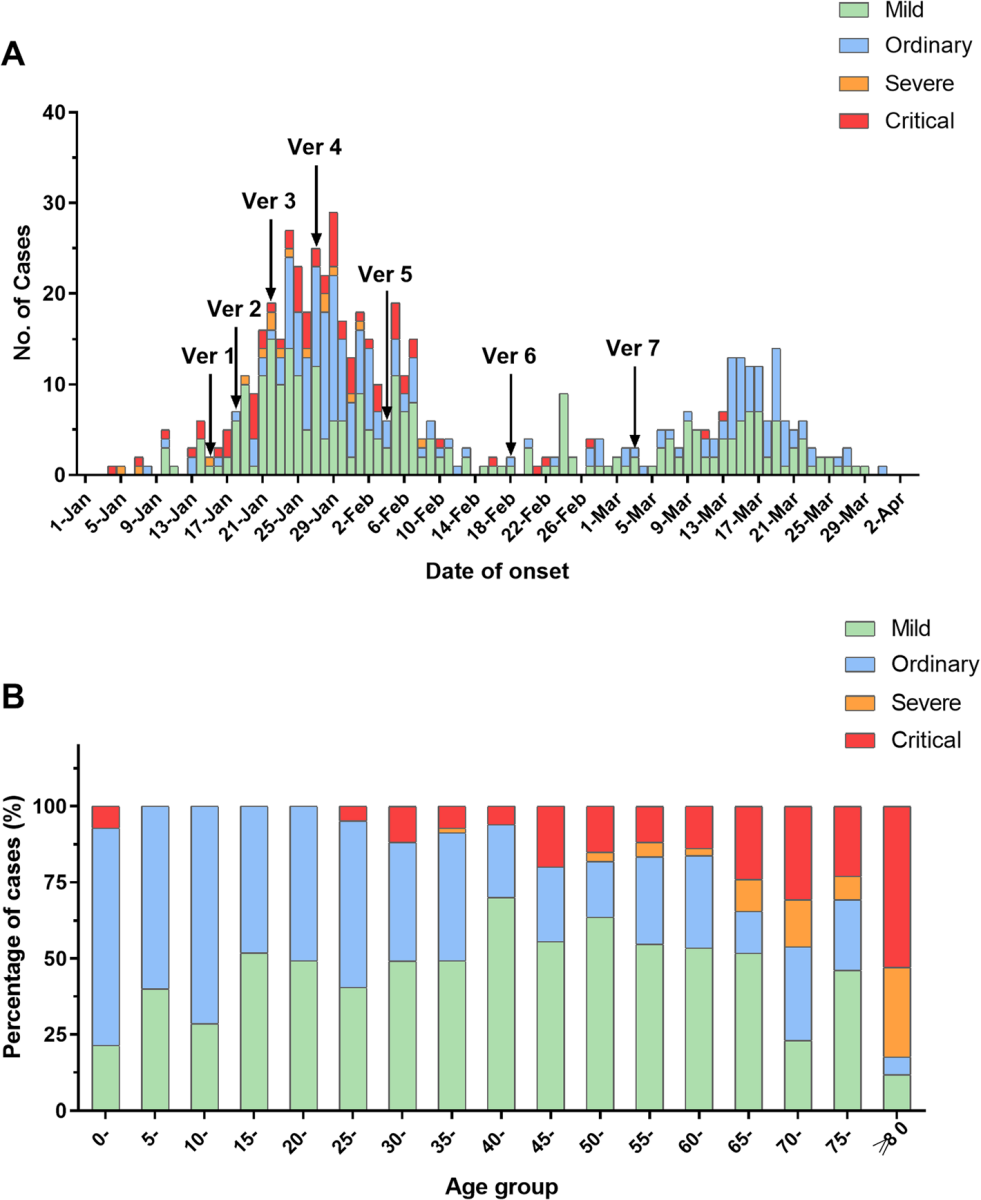
The clinical severity of 585 confirmed case-patients with SARS-CoV-2 infection. Note: Ver: Version. The different version of definition for confirmed cases were marked

Of all confirmed case-patients, 36.3% were mild, 49.7% were moderate, 11.3% (64/585) were severe cases and 2.7% (16/585) were critically ill. The proportion of severe and critical cases decreased from 21.4% before Feb 1st to 7.2% after Feb 1st (Fig. 3a). The association between illness severity and age was shown in Fig. 3b. It was shown that illness severity aggravated with age (Supplementary Table 2, *χ*^2^ = 50.576, *P* < 0.001). A total of 8 cases deceased (with a crude case-fatality rate of 1.4%), among which 7 deaths were elderly adults over 60 years and 1 death was a 50-year-old man. The case fatality rate for males was 1.9% (5/268) and 1.0% (3/317) for females. The case fatality rate for ≥80 age group was 29.4% (5/17). All of the deaths had comorbid conditions, of which 75.0% (6/8) had hypertension or cardiovascular disease. Among 46 children < 18 years, 28 (60.9%) were mild, 17 (37.0%) were moderate and 1 (2.2%) was severe. The severe case was a three-year-old child with leukemia.

### Medical care timelines

The median duration from onset of symptoms to their first medical visit was estimated to be 3 days (P_2.5_-P_97.5_: 0–17). The median duration from onset to case confirmation was estimated to be 5 days (P_2.5_-P_97.5_: 1.0–19.5).

### Incubation estimates

We reviewed the records of the confirmed cases and found 37.4% (219/585) had clear history of contacts with cases prior to symptom onset; based on which we estimated that the mean incubation period was 6.3 days (95% CI: 6.0–6.6) and the median was 5.7 days (P_2.5_-P_97.5_: 5.2–6.1).

### Close contacts

By Apr 3rd, a total of 4007 close contacts were quarantined, 186 were confirmed with SARS-CoV-2 infection, with an overall secondary attack rate of 4.6% (95% CI: 4.0–5.3). The secondary rate was higher among family members or relatives (15.6%, 111/714) than that among social contacts (2.2%, 75/3363) (*χ*^2^ = 239.852, *P* < 0.001).

Among 441 close contact of HCWs, 32 were confirmed with SARS-CoV-2 infection, with an overall secondary attack rate of 7.3%, which was higher than that of non-HCWs’, with a secondary attack rate of 4.2% (154/3636) (*χ*^2^ = 8.243, *P* = 0.004).

### Clusters

**Fig. 4:**
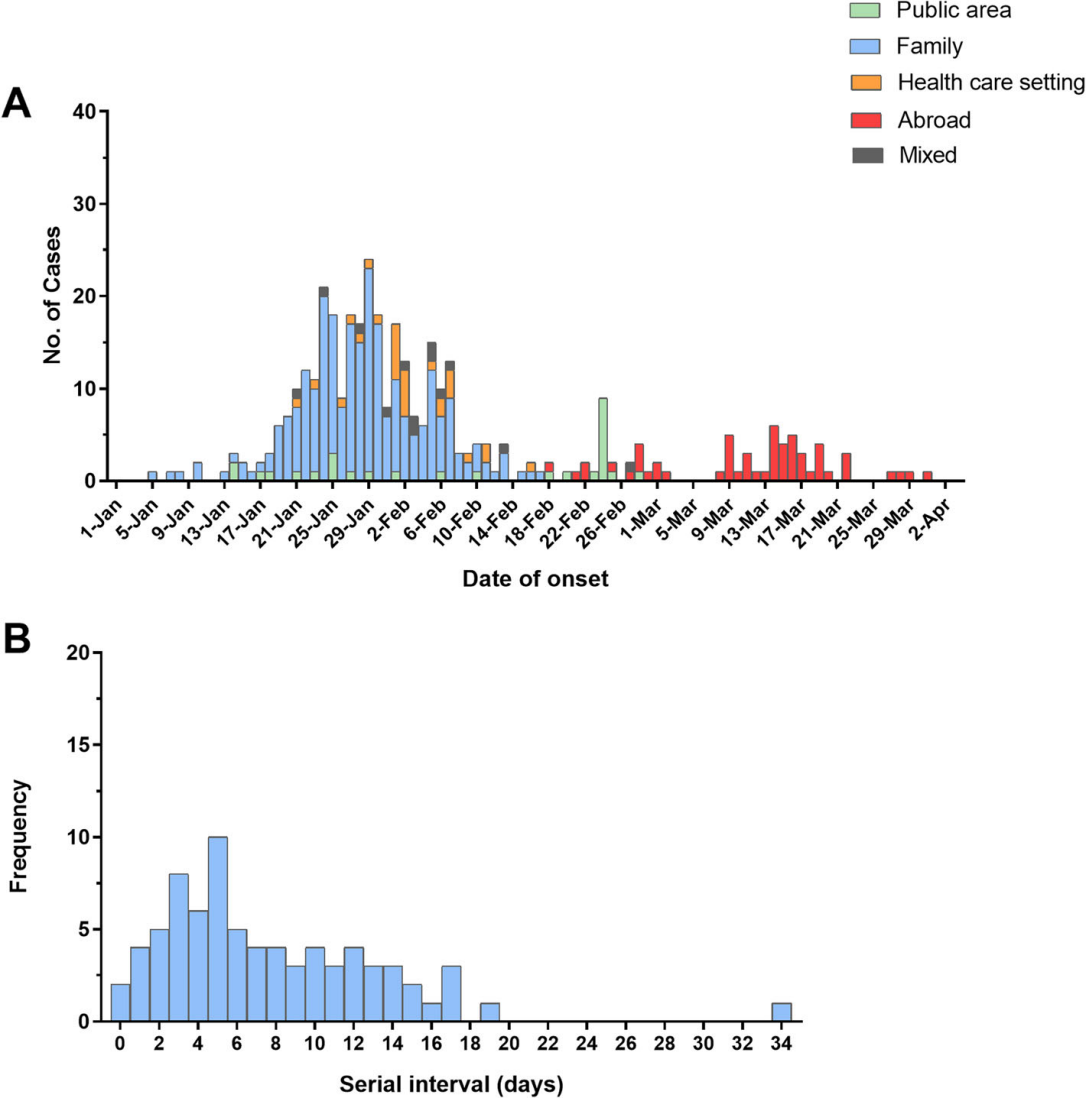
The source and distribution of clusters and the serial interval for family cluster introduced one single index case

Till Apr 3rd, a total of 117 clusters occurred, involving 391 confirmed cases. Among 391 cases, 246 (66.3%) occurred in family, 56 from abroad (15.1%), 28 (7.6%) in health care facilities, 28 (7.6%) in public areas and 13 (3.5%) in mixed areas. Before Feb 18th, clusters mainly occurred in family and then were predominantly from abroad after Feb 28th (Fig. 4a).

There were 246 confirmed cases were involved in 91 family clusters. The median number of involved cases of family clusters was 3 (Range, 2–7; IQR, 2–3). To estimate the basic reproduction number and serial interval in families, we identified 38 family clusters where one single index case was introduced. Since the 38 index cases caused 76 secondary cases among 193 family close contacts, we estimated that *R*_*0*_ in family clusters was 2.0 (95% CI: 1.6–2.4). And the average serial interval was estimated at 7.6 days (95% CI: 6.4–8.9). The median serial interval was 6.0 days (range, 0–34; IQR, 3.3–11.0) (see Fig. 4b).

Two clusters occurred in hospitals, involving 38 confirmed cases. One cluster occurred in the cardiac intensive care unit (CICU) and intensive care unit (ICU) of a general hospital in Beijing, involving 35 cases, among which 7 were HCWs. Another clusters in hospital involved 3 cases including one hospitalized patient and her two relatives providing paramedical assistants.

### SARS-CoV-2 testing among ILI cases

From Jan 28th to Apr 3rd 2020, a total of 3267 specimens were collected from 3267 individuals, and no SARS-CoV-2 viral RNA was identified.

### Viral RNA in clinical samples

Among all 585 confirmed case-patients, 936 specimens from 243 cases were available for analysis, including 612 pharyngeal swabs, 7 nasal swabs, 5 saliva specimens, 219 sputa specimens, 2 serum/plasma specimens, 22 urine specimens, and 69 fecal specimens. 354 pharyngeal swabs, 7 nasal swabs, 156 sputa specimens, and 28 fecal specimens showed positive results in real-time RT-PCR tests. The RNA positive rate of throat swabs in mild, ordinary, severe and critical cases was 58.73, 55.17, 62.07, and 76.47%, respectively (*χ*^2^ = 4.050, *P* = 0.256). In contrast to these, the RNA positive rate of sputum in mild, ordinary, and severe cases was 63.08, 75.86, and 70.27%, respectively (*χ*^2^ = 3.329, *P* = 0.189). No viral RNA was found in all 22 urine specimens, 2 serum/plasma specimens and 5 saliva specimens.

### Phylogenetic analysis of SARS-CoV-2 in Beijing

**Fig. 5:**
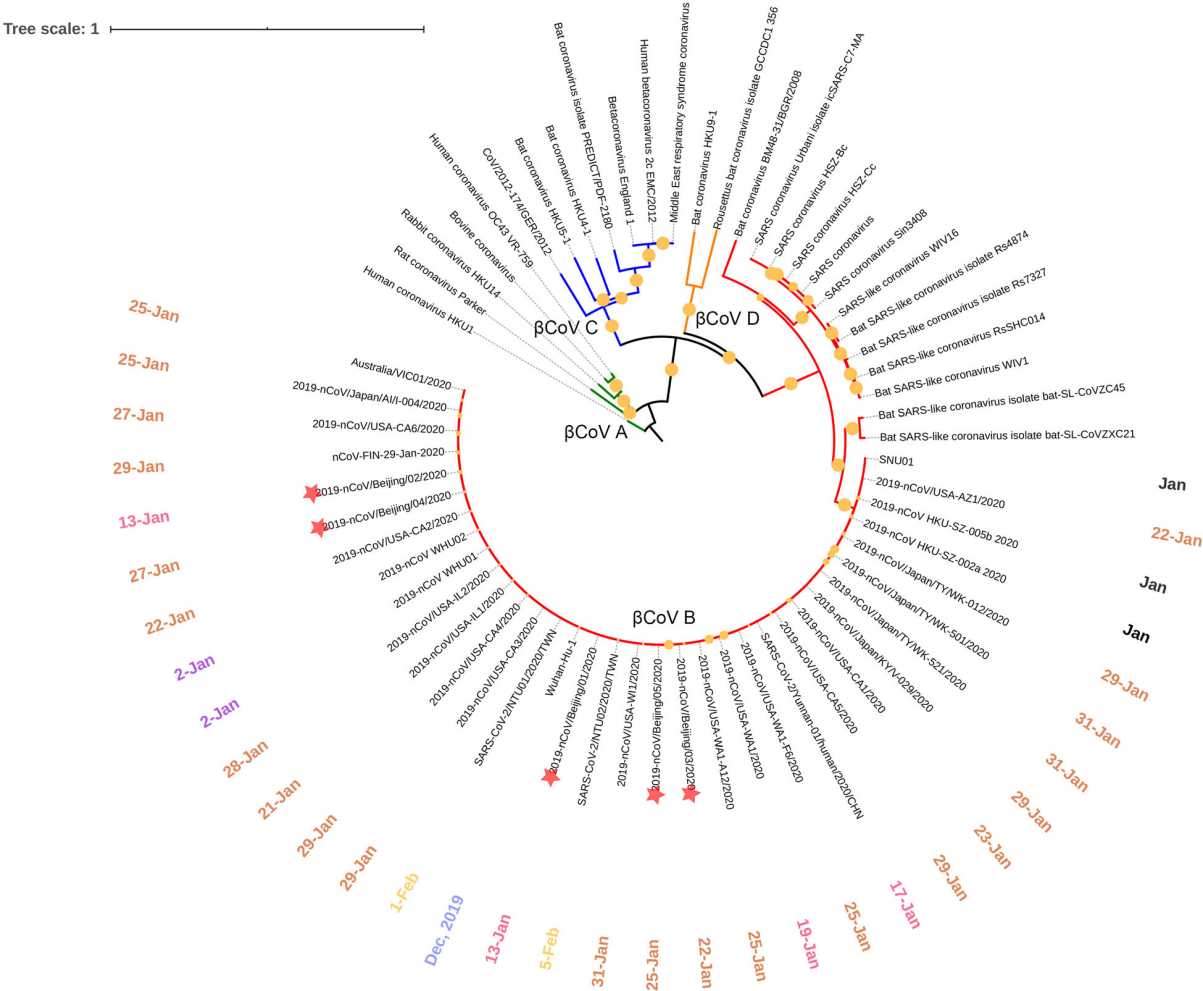
The phylogenetic tree of SARS-CoV-2 in Beijing, China, 2020. The tree was constructed by the N-J method using the HKY model with bootstrap values determined by 1000 replicates. The bootstraps are shown at the branch point. The isolated time was colored at tags

A total of five viral full genomes were obtained during the study period, including four sequences from imported cases from Whuan and one from a secondary case. Phylogenetic analysis suggested all tested viruses belonged to lineage B of the genus beta-coronavirus, and is genetically closely related to SARS-CoV-2 isolates in Wuhan (Fig. 5). It showed high genetic similarity among all tested viruses of 99.97–99.99%. It is worth noting that all five viruses carried 442 L, 472F, 479Q, 487 N, and 491Y in viral S gene receptor-binding subdomain.

## Discussion

The transmission of COVID-19 in Beijing was initially imported from Wuhan, Hubei of China, then generated subsequent community clusters in Beijing, dominated by intrafamilial transmission. Later, after the epidemic was controlled in China, imported cases from abroad were predominantly. Our results could provide important epidemiological and genetic parameters from the real-world settings outside Wuhan for further analysis, including evaluations of the impact of control strategy and predictions of COVID-19 dissemination in large cities. We also showed the importance of enhanced surveillance and testing capacity in a mega-city where a very small proportion of the population are immune to the virus. Until a vaccine is available, this is critical to prevent new epidemics from arising.

We estimated basic epidemiological parameters from family clusters where exposure and infection details were well characterized. We estimated an average serial interval of 7.6 days based on the data of 38 family clusters, which was similar to Li’s [9]. We further bring up the 95% CI from 5.3–19.0 to a higher precision of 6.4–8.9 due to the larger sample size. We also obtained a similar estimate of *R*_*0*_ approximately 2 (95% CI: 1.6–2.4) to Li’s (indicating that each patient was able to further infect 2 persons on average) [9]. Some estimates of *R*_*0*_ used the report date of cases, and are not as reliable as *R*_*0*_ estimates from the date of symptom onset. The *R*_*0*_ for SARS-CoV-2 was similar to SARS. In contrast, the estimated *R*_*0*_ of SARS coronavirus ranged from 1.1 to 4.2 with most estimates between 2 and 3 [10–12]. However, the major difference with COVID 19 is pre- and asymptomatic transmission, which makes the disease control practice far more challenging. The previous models highly relied on the assumptions underpinning the models, the timing of diagnosing and reporting of confirmed cases.

We estimated the secondary transmission of COVID-19 in 2902 close contacts. The secondary attack rate of household was statistically higher than that of social contact (16.6% vs. 3.4%, *P* < 0.001). This suggests that disease control around contact tracing should focus on spread from person to person among household close contacts as the highest risk. Thus, the implementation of prevention and control measures in household is particularly required. The household secondary attack rate of COVID-19 was slightly higher than SARS in Beijing, 2003 [13, 14]. This might imply that the transmissibility of SARS-CoV-2 was higher than SARS. Due to the atypical or unspecific presence of mild infection or asymptomatic infection, the family members had been fully exposed to cases before they were confirmed, and this increased the risk of infection among household close contacts. In addition, we found that the secondary attack rate of HCWs was relative higher than that of non-HCWs’ which implied that we should focus on the prevention among HCWs who might cause potential clusters or outbreaks both in healthcare facilities and families.

On Jan 23rd, the Chinese government officially announced the implementation of lockdown of Wuhan City. We showed that from Feb 1st, about 1 incubation period from the lockdown, the proportion of cases with a travel history to Wuhan progressively declined, indicating the impact of lockdown of Wuhan on the epidemic in Beijing.

The laboratory findings for SARS-CoV-2 infection pointed out the preference of lower respiratory tract samples in real-time RT-PCR. The positive rate of sputum was slightly higher than that of pharyngeal swab in moderate and severe cases. Our previous quantitative study also showed the relatively high viral loads in sputum with a median of 7.52 × 10^5^ [15]. Recent reports also suggested some other types of sample for SARS-CoV-2 RNA testing [16, 17], including nasopharyngeal swabs, saliva samples and tear samples. The performances of these specimens need further assessment since the viral RNA was not found in urine samples and saliva samples in our study.

None of the 3267 samples from ILI cases based on routine ILI surveillance was tested positive for SARS-CoV-2. This provides confidence that COVID-19 has not spread widely in Beijing, China. Little genetic variance was found in SARS-CoV-2 viruses in Beijing. When the virus jumped directly from bats to human or via some unknown intermediate hosts is still unclear [18, 19], the genetic analysis suggested the SARS-CoV-2 had a relatively low estimated mean evolutionary rate of 1.79 × 10^− 3^ to 1.82 × 10^− 3^ substitutions per site per year [20], which was in consistent with our results. In this study, a high similarity was observed in tested strains collected at the early stage of COVID-19 outbreak when compared with reference strain (NC_045512). However, numerous novel mutations, such as S-D614G, were identified in current strains worldwide according to recent studies [21]. The increased genetic diversity of SARS-CoV-2 in human hosts over time cannot be neglected.

Our analysis had several limitations. First, if the close contacts of confirmed cases traveled to other provinces outside Beijing, some of them might be lost to follow-up. This might bring bias to the secondary attack rate of close contact and thus lead to lower *R*_*0*_ estimates. Second, detailed information on such exposure history as frequency, intensity, and duration for all cases was not available. This may influence the estimates of the incubation period. Third, the relationship between index cases and their quarantined close contact was not well recorded, which limited the analysis of secondary attack rates by different relationships. A strength of the study is the well-established surveillance systems such as ILI surveillance, which were able to provide enhanced capability to study the epidemiology and exclude substantial community transmission.

## Conclusion

The transmissibility of SARS-CoV-2 was relatively high, especially among households and from HCWs, which should be focused on. The lockdown of Wuhan City was effective in halting the spread of the COVID-19 to Beijing.

## Supplementary information

**Additional file 1 Supplementary Table 1**. The definitions of suspected case and confirmed cases used in this study. **Supplementary Table 2**. The type of severity of different age group for 585 confirmed cases in Beijing, China

## Data Availability

The datasets used and/or analysed during the current study are available from the corresponding author on reasonable request.

## Abbreviations

CoV: Coronavirus
COVID-19: Coronavirus disease 2019
AI: Artificial intelligence
ILI: Influenza-like illness
RT-PCR: Reverse transcriptase polymerase chain reaction
NGS: Next-generation sequencing
N-J: Neighbor-Joining
CI: Confidential interval
HCWs: Healthcare workers
