# Supplementary material for: Basic epidemiological parameter values from data of real-world in mega-cities: the characteristics of COVID-19 in Beijing, China
Source: BMC Infect Dis. 2020 Jul 20;20:526. doi: 10.1186/s12879-020-05251-9 (PMC7370267; doi:10.1186/s12879-020-05251-9)
Supplement: Supplementary file 1 — Additional file 1 Supplementary Table 1. The definitions of suspected case and confirmed cases used in this study. Supplementary Table 2. The type of severity of different age group for 585 confirmed cases in Beijing, China [file 12879_2020_5251_MOESM1_ESM.docx]

**Supplementary Table 1 The definitions of suspected case and confirmed cases used in this study**

| Date |  | 15 Jan to 17 Jan | 18 Jan to 21 Jan | 22 Jan to 26 Jan | 27 Jan to 3 Feb | 4 Feb to 17 Feb | 18 Feb to 2 Mar | After 3 Mar |
| --- | --- | --- | --- | --- | --- | --- | --- | --- |
| Version |  | 1 | 2 | 3 | 4 | 5 | 6 | 7 |
| Suspected Cases | Epidemiology manifestations | Having a history of travel in Wuhan, or contact with Huanan Seafood Wholesale Market within 14 days before the patient’s onset; | (1) Having a history of travel or residence in Wuhan within 14 days before the patient’s onset; | (1) Having a history of travel or residence in Wuhan or other communities with cases reported within 14 days before the patient’s onset; | (1) Having a history of travel or residence in Wuhan and its surrounding areas or other communities with cases reported within 14 days before the patient’s onset; | (1) Having a history of travel or residence in Wuhan and its surrounding areas or other communities with cases reported within 14 days before the patient’s onset; | (1) Having a history of travel or residence in Wuhan and its surrounding areas or other communities with cases reported within 14 days before the patient’s onset; | (1) Having a history of travel or residence in Wuhan and its surrounding areas or other communities with cases reported within 14 days before the patient’s onset; |
|  |  |  | (2) Having a contact history with patients with fever or respiratory symptoms from Wuhan within 14 days before the patient’s onset; | (2) Having a contact history with patients with fever or respiratory symptoms from Wuhan, or the communities with cases reported within 14 days before the patient’s onset; | (2) Having a contact history with patients (a positive results of nucleic acid test of 2019-nCoV) within 14 days before the patient’s onset; | (2) Having a contact history with patients (a positive results of nucleic acid test of 2019-nCoV) within 14 days before the patient’s onset; | (2) Having a contact history with patients (a positive results of nucleic acid test of 2019-nCoV) within 14 days before the patient’s onset; | (2) Having a contact history with patients (a positive results of nucleic acid test of 2019-nCoV) within 14 days before the patient’s onset; |
|  |  |  | (3) Clustering occurrence of cases or a link with confirmed cases. | (3) Clustering occurrence of cases or a link with confirmed cases, mild cases or asymptomatic cases. | (3) Having a contact history with patients with fever or respiratory symptoms from Wuhan and its surrounding areas, or the communities with cases reported within 14 days before the patient’s onset; | (3) Having a contact history with patients with fever or respiratory symptoms from Wuhan and its surrounding areas, or the communities with cases reported within 14 days before the patient’s onset; | (3) Having a contact history with patients with fever or respiratory symptoms from Wuhan and its surrounding areas, or the communities with cases reported within 14 days before the patient’s onset; | (3) Having a contact history with patients with fever or respiratory symptoms from Wuhan and its surrounding areas, or the communities with cases reported within 14 days before the patient’s onset; |
|  |  |  |  |  | (4) Clustering occurrence of cases. | (4) Clustering occurrence of cases (two or more patients with fever or respiratory symptoms in one place within two weeks). | (4) Clustering occurrence of cases. | (4) Clustering occurrence of cases (two or more patients with fever or respiratory symptoms in one place within two weeks). |
|  | Clinical Manifestations | (1) Fever; | (1) Fever; | (1) Fever; | (1) Fever and/or respiratory symptoms; | (1) Fever and/or respiratory symptoms; | (1) Fever and/or respiratory symptoms; | (1) Fever and/or respiratory symptoms; |
|  |  | (2) Having the imaging features of pneumonia; | (2) Having the imaging features of pneumonia; | (2) Having the imaging features of pneumonia; | (2) Having the imaging features of pneumonia; | (2) Having the imaging features of COVID-19 pneumonia; | (2) Having the imaging features of pneumonia described above; | (2) Having the imaging features of pneumonia described above; |
|  |  | (3) In the early stage, a normal or decreased total white blood cell count and a decreased lymphocyte count can be found; | (3) In the early stage, a normal or decreased total white blood cell count and a decreased lymphocyte count can be found. | (3) In the early stage, a normal or decreased total white blood cell count and a decreased lymphocyte count can be found. | (3) In the early stage, a normal or decreased total white blood cell count and a decreased lymphocyte count can be found. | (3) In the early stage, a normal or decreased total white blood cell count and a decreased lymphocyte count can be found. | (3) In the early stage, a normal or decreased total white blood cell count and a decreased lymphocyte count can be found. | (3) In the early stage, a normal or decreased total white blood cell count and a decreased lymphocyte count can be found. |
|  |  | (4) No reduction in symptoms after antimicrobial treatment for 3 days. |  |  |  |  |  |  |
|  | Criteria | Patients who satisfy the epidemiological exposures histories as well as all three clinical manifestations can be diagnosed as suspected cases. | Patients who satisfy any one of the epidemiological exposures histories as well as all three clinical manifestations can be diagnosed as suspected cases. | Patients who satisfy any one of the epidemiological exposures histories as well as all three clinical manifestations can be diagnosed as suspected cases. | Patients who satisfy any one of the epidemiological exposures histories as well as any two of the clinical manifestations can be diagnosed as suspected cases. Patients with no definite epidemiological history can be diagnosed only if all the three clinical manifestations are met. | Patients who satisfy any one of the epidemiological exposures histories as well as any two of the clinical manifestations can be diagnosed as suspected cases. Patients with no definite epidemiological history can be diagnosed only if all the three clinical manifestations are met. | Patients who satisfy any one of the epidemiological exposures histories as well as any two of the clinical manifestations can be diagnosed as suspected cases. Patients with no definite epidemiological history can be diagnosed only if all the three clinical manifestations are met. | Patients who satisfy any one of the epidemiological exposures histories as well as any two of the clinical manifestations can be diagnosed as suspected cases. Patients with no definite epidemiological history can be diagnosed only if all the three clinical manifestations are met. |
| Confirmed cases | Etiological evidences | (1) The virus gene sequence is highly homologous to the known 2019-nCoV by NGS in respiratory tract specimens. | (1) A positive result of the nucleic acid of 2019-nCoV in respiratory tract specimen and blood specimen by real-time RT-PCR. | (1) A positive result of the nucleic acid of 2019-nCoV in respiratory tract specimen and blood specimen by real-time RT-PCR. | (1) A positive result of the nucleic acid of 2019-nCoV in respiratory tract specimen and blood specimen by real-time RT-PCR. | (1) A positive result of the nucleic acid of 2019-nCoV by real-time RT-PCR. | (1) A positive result of the nucleic acid of 2019-nCoV by real-time RT-PCR; | (1) A positive result of the nucleic acid of 2019-nCoV by real-time RT-PCR; |
|  |  |  | (2) The virus gene sequence is highly homologous to the known 2019-nCoV. | (2) The virus gene sequence is highly homologous to the known 2019-nCoV. | (2) The virus gene sequence is highly homologous to the known 2019-nCoV in respiratory tract specimen and blood specimen. | (2) The virus gene sequence is highly homologous to the known 2019-nCoV. | (2) The virus gene sequence is highly homologous to the known 2019-nCoV. | (2) The virus gene sequence is highly homologous to the known 2019-nCoV. |
|  | Criteria | The suspected cases with the etiological evidence can be diagnosed as confirmed cases. | The suspected cases with one of the following etiological evidences can be diagnosed as confirmed cases. | The suspected cases with one of the following etiological evidences can be diagnosed as confirmed cases. | The suspected cases with one of the following etiological evidences can be diagnosed as confirmed cases. | The suspected cases with one of the following etiological evidences can be diagnosed as confirmed cases. | The suspected cases with one of the following etiological evidences can be diagnosed as confirmed cases. | The suspected cases with one of the following etiological evidences can be diagnosed as confirmed cases. |

**Supplementary Table 2 The type of severity of different age group for 585 confirmed cases in Beijing, China**

| Age group | Mild (%) | Moderate (%) | Severe (%) | Critical (%) | Total (%) |
| --- | --- | --- | --- | --- | --- |
| 0-5 | 12 (5.66) | 3 (1.03) | 1 (1.52) | 0 (0) | 16 (2.74) |
| 6-17 | 16 (7.55) | 14(4.81) | 0 (0) | 0 (0) | 30 (5.13) |
| 18-59 | 159 (75.00) | 227 (78.01) | 37 (56.06) | 4 (25.00) | 427 (72.99) |
| 60- | 25 (11.79) | 47 (16.15) | 28 (42.42) | 12 (75.00) | 112(20.85) |
| Total | 212 (100.00) | 291(100.00) | 66 (100.00) | 16 (100.00) | 585 (100.00) |
